# Evaluation of the efficacy and safety of artemether emulsion on localized senile pruritus: A randomized pilot study

**DOI:** 10.1097/MD.0000000000030472

**Published:** 2022-09-02

**Authors:** Hui-Qiong He, Wen-Tong Shen, Qin Pei, Jian-Biao Fei, Yue Yu, Hai-Hong Qin, Guo-Jiang Wang

**Affiliations:** a Department of Dermatology, Shanghai University of Medicine and Health Science Affiliated Zhoupu Hospital, Shanghai, China.

**Keywords:** artemether, efficacy, elderly, localized senile pruritus

## Abstract

Localized senile pruritus is a continued health problem for the elderly. This study aimed to evaluate the efficacy and safety of artemether emulsion on localized senile pruritus.

**Methods:**

Sixty patients diagnosed with senile pruritus were randomized into the artemether emulsion (1%) group or emulsion base group in a 1:1 ratio (the artemether group vs the control group). The patients used artemether emulsion or emulsion base for pruritus twice daily for 2 weeks. The pruritus visual analog scale (VAS) and the rate of adverse events were evaluated in week 0 and week 2.

**Results:**

The VAS scores in week 2 after treatment decreased significantly compared with those before treatment in both groups (*P* < .05). After treatment, patients receiving the artemether emulsion had significantly lower mean VAS scores compared to those who received the emulsion base (1.21 ± 1.64 vs 3.67 ± 2.97, *P *< .05). When the VAS scores were compared between the 2 groups before treatment, the effective rate of the artemether group was significantly higher than that of the control group (*χ*^2^ = 55, *P* < .05) in week 2 after treatment. Besides, no adverse events occurred in both groups.

**Conclusions:**

Both artemether emulsion and emulsion base were effective in treating localized senile pruritus, and artemether emulsion was superior to emulsion base.

## 1. Introduction

Senile pruritus is a common dermatological disease in the elderly aged over 65 years and usually occurs in the autumn and winter.^[1,2]^ Itching can involve the whole body or part of the body in elderly patients and leads to poor quality of life.^[1,2]^ The main treatment for localized senile pruritus is topical drugs, including moisturizing emulsions, calcineurin inhibitors, and glucocorticoid emulsions.^[1,2]^ Moisturizers are inexpensive and suitable for asteatosis and mild pruritus, which can improve the patient’s condition through frequent moisturization.^[3]^ Calcineurin inhibitors are effective for atopic dermatitis or neurodermatitis with few adverse hormonal reactions; however, some patients may develop irritant dermatitis.^[1,4]^ Glucocorticoids have good antipruritic effects; however, long-term application can cause side effects, such as skin atrophy, telangiectasia, or hypersensitivity.^[5,6]^ Therefore, exploring new topical drugs for treating senile pruritus is of great clinical significance.

Artemisinin is derived from the traditional Chinese herb “Artemisia” and has anti-inflammatory and anti-allergic effects. Artemisinin-type compounds have been widely used for treating solar dermatitis, psoriasis, lupus erythematosus, rosacea-like dermatitis, and other skin diseases.^[7,8]^ Artemether, a derivative of artemisinin, has been shown to improve inflammation and allergy.^[9,10]^ In recent reports, artemether emulsion was found to have an antipruritic effect in the treatment of rosacea^[11]^ and acne^[12]^ with few side effects.

Herein, we aimed to investigate the efficacy and safety of artemether emulsion (1%) for treating localized senile pruritus.

## 2. Methods

### 2.1. Study design

This was a randomized pilot study. The study was approved by the institutional ethical review board of the Shanghai University of Medicine and Health Science Affiliated Zhoupu Hospital (2022-C-024-E01), and all participants provided written informed consent.

Patients recruited from the Department of Dermatology of our hospital were randomized into the artemether emulsion (1%) group or the emulsion base group in a 1:1 ratio, following the random number table by a blinded researcher. Artemether emulsion or emulsion base was administered twice daily for 2 weeks to each patient. The study researchers instructed each patient to use the drug according to the standard procedure. The first patient was enrolled on September 2, 2021, and the last patient completed the 2-week treatment on April 27, 2022. The study evaluation time points were baseline and week 2.

### 2.2. Patients

The patients included were aged over 60 years, had a pruritus area of <10% of the body surface area, and had to meet the diagnostic criteria of pruritus (no primary skin lesion but may have secondary skin lesions, such as scratches, blood scabs, pigmentation, etc).^[13]^ The exclusion criteria were as follows: generalized pruritus or a pruritus area of >10% of the body surface area, requiring systemic drugs; known allergies to any component of the formula in artemether emulsion; patients with neurological or mental diseases; patients treated with glucocorticoids or immunosuppressants within 1 month before inclusion; lactating women and those with immune deficiency, local infection, or previous history of skin cancer in the affected areas; patients who received oral antihistamines within 2 weeks or other emulsions within 1 week; patients with severe heart, liver, or kidney disease, diabetes, tumors, or other immunocompromised diseases; and patients unable to follow the doctor’s treatment instructions or those who were lost to follow-up.

### 2.3. Study procedures and evaluations

At the time of recruitment, baseline patient information, including sex, age, and course, was recorded. All participants who completed the treatment underwent further evaluation of the outcomes in week 2.

Treatment efficacy was assessed using the pruritus visual analog scale (VAS).^[14]^ The methods were as follows: the patient was asked to mark using a 10-cm scale the location where they most experienced itchiness in the past 24 hours, and the physician recorded the score according to the location marked by the patient. The scores and corresponding indicators were as follows: 0, no itching; 1 to 3, mild itching; 4 to 6, moderate itching; 7 to 10, severe itching. The assessment was performed at baseline and in week 2. The efficacy index (*I*) was calculated according to the VAS score as follows: *I* = [(baseline score – week 2 score) ÷ baseline score] × 100%, where *I* = 100% indicates remission, 70% ≤ *I* < 100% indicates marked improvement, 30% ≤ *I* < 70% indicates moderate improvement, and *I* < 30% indicates no improvement. *I* = 70% ≤ *I* ≤ 100% was defined as the effective rate, which included remission and marked improvement in the participants.

Treatment safety included systemic and local adverse events were determined by the patients and physicians based on local tolerance parameters (stinging/burning, dryness, and itching) on a 4-point scale (from 0 [none] to 3 [severe]) during the 2 weeks.

### 2.4. Statistical analysis

SPSS 22.0 software (Armonk, NY) was used for statistical analysis. Nonparametric tests were used for comparing the VAS scores before and after treatment (data not conforming to normal distribution), and the chi-square test was used for comparing efficacy between groups. Statistical significance was set at *P* < .05.

## 3. Results

### 3.1. Baseline data

The number of randomly assigned patients in the 2 groups was 30. In week 2, 2 cases in the artemether emulsion group and 3 in the emulsion base group were lost to follow-up.

The baseline characteristics were balanced between the 2 groups (Table 1). The average age was 71.7 years in the artemether emulsion group and 70.5 years in the emulsion base group, and the proportion of females was 57% and 52%, respectively (*P* > .05). The average course was 14.1 months for both groups (*P* > .05).

**Table 1:** Baseline demographic and disease characteristics.

	Artemether emulsion	Emulsion base	Value
	(n = 28)	(n = 27)	
Male/female	12/16	13/14	*P* = .51
Mean age (years, mean ± standard deviation)	71.7 (60–83)	70.5 (61–84)	*t* = 0.72
Average course (months)	14.1 (1–60)	14.1 (1–58)	*P* = .66

### 3.2. Efficacy of artemether emulsion

**Figure 1.**
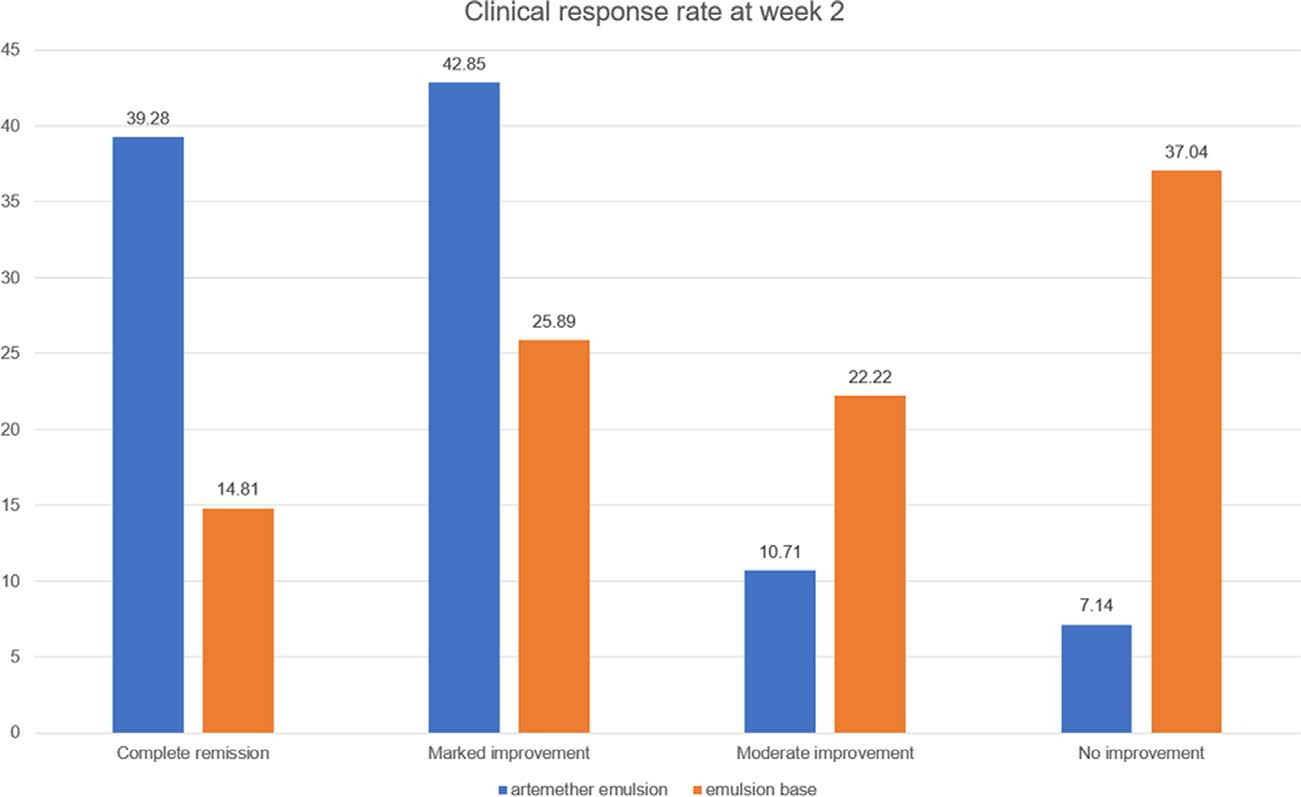
Clinical response rate in week 2.

The mean baseline VAS scores were comparable between the artemether emulsion and emulsion base groups (6.82 ± 0.98 vs 6.93 ± 1.11, *P* > .05). In week 2, the VAS scores in both groups decreased significantly compared to the baseline VAS scores (both *P* < .05, Table 2). Patients receiving artemether emulsion had significantly lower mean VAS scores than those who received the emulsion base (1.21 ± 1.64 vs 3.67 ± 2.97, *P* = .001, Table 2). The effective response rate in week 2 was significantly higher in the artemether emulsion group than in the emulsion base group (82.13% vs 40.70%, *P *= .013, Fig. 1). No adverse reactions occurred in either group of patients during the treatment period.

**Table 2:** Visual analog scale scores at baseline and in week 2 (mean ± standard deviation).

	Baseline	Week 2	Z	*P* value
Artemether emulsion	6.82 ± 0.98	1.21 ± 1.64	–6.327	<.01
Emulsion base	6.93 ± 1.11	3.67 ± 2.97	–4.018	<.01
*Z*	–0.15	–3.3		
*P*	0.88	0.001		

## 4. Discussion

In this study, artemether emulsion (1%) significantly improved localized senile pruritus compared to the emulsion base without adverse events. This demonstrates the effectiveness of artemether in the treatment of senile pruritus.

Senile pruritus often has an unknown etiology and decreases the quality of sleep and life.^[1,2]^ Thus, senile pruritus often debilitates patients.^[3]^ The pathogenesis of senile pruritus has not been fully elucidated. Recent studies have shown that the disease is related to the deterioration of skin barrier function, aging of the immune system, inflammatory aging, degenerative changes of the nervous system, changes in endocrine hormones, etc.^[1–3]^ When the epidermal barrier function is impaired, external allergens can easily invade the epidermis and cause the occurrence of atopic dermatitis.^[4,15]^ The body then stimulates the production of pro-inflammatory factors to repair the damaged skin barrier.^[15]^ However, the immune function of elderly patients exhibits synchronous senescence, which is defined as “inflamm-aging.”^[16,17]^ Inflammatory aging refers to a chronic and low-grade inflammatory state manifested by increased expression of pro-inflammatory factors such as interleukin (IL)-1, IL-6, IL-8, IL-18, tumor necrosis factor-alpha, interferon (IFN)-alpha, IFNβ, and monocyte chemoattractant protein-1.^[17]^ Chronic pruritus is associated with the high expression of various pruritus inflammatory mediators, including IL-2, IL-6, IL-31, IL-4, IL-13, INF-γ, etc.^[18]^ Previous studies have shown that the secretion of these pro-inflammatory factors in senescent cells is mainly regulated by the nuclear factor kappa-light-chain-enhancer of activated B cell (NF-κB) signaling pathway.^[19]^ High NF-κB expression promote the expression of several pro-inflammatory factors.

Artemisinin and its derivatives inhibit NF-κB signaling activity by blocking several upstream NF-κB signaling pathways, resulting in the downstream inhibition of a series of activities, including the release of pro-inflammatory factors.^[7]^ As a derivative of artemisinin, artemether can improve the inflammatory state in rheumatoid arthritis, diabetes, neuritis, and other diseases, mainly by inhibiting the expression of IL-2, IL-6, IFNγ, and other itchy inflammatory mediators.^[9,10,20–22]^ Our study confirmed the anti-inflammatory effects of artemether.

Topical ointments, including moisturizing emulsions, glucocorticoid emulsions, and calcineurin inhibitors (tacrolimus and pimecrolimus creams), are mainly used for treating localized pruritus.^[1–6]^ Local and external use of glucocorticoid emulsions can reduce desmosome density in the lower cuticle and destroy the keratinoid lipid membrane, resulting in the destruction of skin integrity and increased transdermal water loss.^[23,24]^ Therefore, it is not recommended for use in elderly patients with inflammatory aging. Moisturizing creams can repair the skin barrier and reduce transdermal water loss but have no anti-inflammatory effect.^[3]^ Therefore, moisturizers can be used in combination with other creams that have anti-inflammatory effects. Previous reports have shown the key role of moisturizers in relieving itching.^[25,26]^ Calcineurin inhibitors such as tacrolimus and pimecrolimus have anti-inflammatory and immunomodulatory effects while treating pruritus simplex.^[1,4,27–29]^ However, tacrolimus and pimecrolimus are relatively expensive and are prone to local irritation and burning sensation in the skin with age-related barrier damage, which may lead to irritant dermatitis or even skin malignancies.^[27–29]^ Therefore, dermatologists are still striving to find effective alternative therapies, such as Chinese herbal preparations and massage therapy.^[27]^

This study showed that artemether cream for local pruritus had an effective rate of 82.1% and had a good effect on localized senile pruritis. There were no obvious adverse reactions after topical application, which provided a new therapeutic option for treating senile pruritus. Its mechanism may be related to the inhibition of pro-inflammatory factor secretion, the NF-κB signaling pathway, and downregulation of pruritus inflammatory mediators by artemether. The specific mechanism will be studied further by a research group.

The limitation of this study was that the sample size was small, and it was a single-center study. More multicenter studies with large sample sizes should be conducted in the future. Moreover, this study did not compare the efficacy of different concentrations of artemether emulsions for senile localized pruritus, which might be a goal of future research.

## 5. Conclusion

Artemether emulsion has a better effect in improving localized senile pruritus compared to emulsion base and does not cause side effects.

## Author contributions

**Conceptualization:** Hui-qiong He, Guo-jiang Wang.

**Data curation:** Hui-qiong He, Wen-tong Shen, Qin Pei, Yue Yu, Hai-hong Qin.

**Formal analysis:** Wen-tong Shen, Jian-biao Fei.

**Investigation:** Hui-qiong He, Qin Pei, Jian-biao Fei, Yue Yu.

**Methodology:** Hui-qiong He, Qin Pei, Jian-biao Fei, Hai-hong Qin, Guo-jiang Wang.

**Project administration:** Hai-hong Qin.

**Resources:** Yue Yu, Hai-hong Qin.

**Supervision:** Guo-jiang Wang.

**Validation:** Hui-qiong He, Jian-biao Fei, Guo-jiang Wang.

**Writing – original draft:** Hui-qiong He, Wen-tong Shen.

**Writing – review & editing:** Wen-tong Shen, Qin Pei, Jian-biao Fei, Yue Yu, Hai-hong Qin, Guo-jiang Wang.

## Correction

When originally published, the funding information in the footnote appeared incorrectly as “This work was supported by the Scientific Research Project of the Shanghai University of Medicine and Health Science Affiliated Zhoupu Hospital (No. ZPXM-2019A-13)” and has been corrected to “This work was supported by the Scientific Research Project of the Shanghai University of Medicine and Health Science Affiliated Zhoupu Hospital (No. ZPXM-2019A-13), and Traditional Chinese Medicine science and technology innovation Project of Pudong New Area, Shanghai (PDZY-2022-090).”
